# HPV vaccination in community health organizations: what is going right and how can it be replicated?

**DOI:** 10.1007/s10552-025-02040-0

**Published:** 2025-07-28

**Authors:** Keely Ulmer, Kristin Lyon-Scott, Ngoc Wasson, Taona P. Haderlein, Amanda Bruegl

**Affiliations:** 1 Department of Obstetrics and Gynecology, Division of Gynecologic Oncology, University of Iowa Hospitals and Clinics Holden Comprehensive Cancer Center, Iowa City, USA; 2 OCHIN, Inc., PO Box 5426, Portland, OR USA; 3 Department of Anesthesiology and Perioperative Medicine, School of Medicine, Oregon Health and Science University, Portland, OR USA; 4 Department of Obstetrics and Gynecology, Oregon Health and Science University, Portland, OR USA

**Keywords:** Human papillomavirus, Infection, HPV vaccination, Cancer, Vaccination rates

## Abstract

**Purpose:**

Human papillomavirus (HPV) vaccination is recommended for individuals between 9 and 12 years of age to prevent six different cancers. Lower rates of vaccination exist among underserved populations. We evaluate rates of up-to-date (UTD) HPV vaccination within a nationwide network representing many medically underserved communities.

**Methods:**

This study was conducted using OCHIN, a diverse national database of over 6 million publicly or underinsured patients seen at an OCHIN clinic from January 2015 to December 2023. HPV vaccination initiation and completion rates were the primary outcomes.

**Results:**

1,848,813 patients were included. HPV vaccination rates for all races and ethnic groups were below the Healthy People 2030 goal of 80.0%. Vaccination varied by race/ethnicity, with Hispanic individuals having the highest rates of UTD vaccination. There was no statistically significant difference between male and female genders for HPV vaccination. Odds of successful completion of the vaccine series were highest when series initiation occurred at ages 9–10 and among those with at least two clinical visits per year.

**Conclusions:**

HPV vaccination completion rates remain below the national goal among all ages and racial/ethnic groups in this large, nationwide cohort though all rates increased throughout the study period for most groups. Hispanic race, younger age at initiation, and higher number of clinical visits had increased odds of HPV vaccination. Notably, the gap between males and females closed. Exploration in how these clinics is appealing to the Hispanic population; caregivers of younger children and the male population should be investigated.

## Introduction

**Fig. 1:**
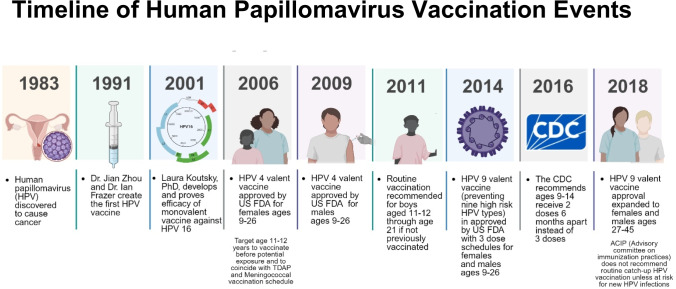
Overview of HPV and HPV vaccination history

High-risk human papillomavirus (HPV) is associated with six different types of cancer: cervical, vaginal, vulvar, anal, penile, and oropharyngeal resulting in an annual 35,000 incident cases in the United States each year [1, 2]. An estimated 79 million Americans are infected with at least one type of HPV, with 14 million new infections occurring each year, nearly half occurring in people aged 14 to 25 years of age [3]. Since the initial identification of HPV and its association with these cancers, highly effective vaccines have been developed (Fig. 1) [4–8].

The 9-valent HPV vaccine protects against 7 of the 14 known high-risk HPV subtypes, 2 of the low-risk HPV subtypes, associated with genital warts, and is Food and Drug Administration (FDA) approved for individuals 9–45 years of age [9]. The Advisory Committee on Immunization Practices (ACIP) and United States Preventive Service Task Force (USPSTF) recommend routine HPV vaccination with a two-dose series for all genders ages 11–12 [10, 11]. The American Cancer Society (ACS) recommends beginning HPV vaccination at age 9 for all genders as this has been shown to increase the probability of vaccine completion by age 13 [2, 5–7]. For individuals who do not receive the vaccine at the recommended time, there is a catch-up period between ages 15 and 26 years with a 3-dose series. For individuals ages 27–45, ACIP recommends against routine vaccination if not previously vaccinated. [8, 9, 11]

The Healthy People 2030 goal for HPV vaccination series completion is 80.0%, however, HPV vaccination for adolescents ages 13–15 remains low at 58.5% based on National Institutes of Health (NIH) data from 2021, and only 38.6% of children ages 9–17 received one or more vaccine doses in 2022 [12]. Evidence also shows that a significant proportion of the US population defers HPV vaccination to the catch-up phase (ages 15–26), which is a period when HPV vaccination is known to be less effective due to increased probability of exposure to the virus [13, 14]. According to the Center for Disease Control and Prevention (CDC), the probability of being vaccinated against HPV in the United States increases with age, is more common in girls than boys, and is more likely in Non-Hispanic White (NHW) populations. It is also more likely among children with private health insurance, and for those living in Metropolitan areas compared to non-metropolitan areas [15].

In this study, we sought to evaluate the rates of HPV vaccine initiation and completion in the OCHIN network, a large clinic network that provides care to systemically underserved communities. The demographics of patients served by this network are primarily Medicaid or uninsured (54% and 18%, respectively), culturally diverse with 23% identifying as people of color and 36% as Hispanic ethnicity, and over half of the patients live under the federal poverty level. Thus, the OCHIN database represents one of the most comprehensive sources of primary healthcare outcomes for traditionally underserved groups in the US and those at elevated risk for the development of future HPV-related cancers. Thus, individuals in this population are ideal for primary prevention through HPV vaccination. Our objective is to examine rates of HPV vaccine series initiation and completion within the OCHIN database to evaluate if primary prevention efforts align with national HPV vaccination goals.

## Methods

### Study design and data source

This cross-sectional study utilized data sourced from the OCHIN Epic electronic health record (EHR) network. As a national nonprofit health IT consultancy, OCHIN provides a shared EHR platform used by more than 34,500 providers and includes over 6 million people in medically underserved communities nationwide. The study was approved by the Oregon Health & Science University Institutional Review Board and follows the Strengthening the Reporting of Observational Studies in Epidemiology (STROBE) reporting guidelines (Institutional Review Board #26,254, March 6, 2024).

### Population

The study population consisted of all persons ages 9–26 years with at least one in-person or telehealth encounter at an OCHIN clinic between 1 January 2015 and 31 December 2023 (*N* = 1,848,813). Exclusion criteria included not visiting an OCHIN clinic for 5 or more years during the study period.

### Data measures and study variables

#### Sociodemographic and clinical characteristics

We obtained sociodemographic patient characteristic information and clinical characteristics including healthcare organization data. Characteristics used to describe our study population include sex, age (9–12 years, 13–18 years, 19–26 years) on January 1 of the first year of meeting inclusion criteria, rurality based on the Rural–Urban Commuting Area (RUCA) code (urban, large rural town, isolated small rural town, missing), number of yearly encounters (< 1, 1, 2–3, 4–7, 8 +), income as a percentage of federal poverty level (FPL) across all encounters (always <  = 138%, always > 138%, over and under 138%, unknown), and insurance status (always insured, always uninsured, mixed). The assigned health system was defined as the healthcare organization the patient had the most encounters with.

#### Exposure and outcomes

Our independent variable was race and ethnicity and it was categorized as Hispanic for those patients who self-reported being Hispanic except for the American Indian and Alaska Natives (AI/AN) patients. Due to relatively small number of AI/AN patients, we did not distinguish between ethnicities within this group. Therefore, the AI/AN category includes people from all ethnicities (Hispanic, non-Hispanic, or unknown). Patients not classified as Hispanic were categorized according to self-reported race information into one of the following groups: non-Hispanic (NH) Asian, NH Black, NH Native Hawaiian/Other Pacific Islander (NHPI/OPI), NH Other/Multi-Race, NH White, and unknown.

The two primary outcomes of our study were as follows: 1. Initiation of the HPV vaccination series and 2. Completion of the vaccination series. HPV vaccination records were collected from immunization, procedure, and health maintenance data within the EHR. Individuals who received their first HPV dose before the age of 15 required only 2 doses to complete the series. Those who started the series at age 15 or older needed 3 doses to be considered fully vaccinated. In addition, a comparison of up-to-date meningococcal and Tdap vaccination to HPV initiation in children 11–12 years old was performed.

### Statistical analysis

Descriptive statistics were used to summarize patient characteristics both overall and by race and ethnicity group, with frequencies and percentages reported. Chi-squared tests assessed the statistical significance differences between groups. We calculated the unadjusted prevalence of HPV initiation and completion by race and ethnicity, age within each racial/ethnic group, and rurality within each racial/ethnic group. The Healthy People 2030 benchmark was included for comparison.

An Analysis of Variance (ANOVA) was conducted to compare the mean HPV initiation rates with those of meningococcal and Tdap vaccinations among children ages 11–12 years. To examine the relationship between race/ethnicity and HPV initiation and completion, we employed a Generalized Estimating Equation (GEE) model with a logit link. This model estimated the odds ratios and 95% confidence intervals, adjusting patient-level characteristics. The GEE model accounted for clustering by assigned health system and adjusted for within-subject correlation due to repeated measurements. Individuals identifying as Hispanic were used as the reference group for race/ethnicity, because this group had the largest sample size. Reference groups for added variables were year 2015, age 9–12, female sex, urban rurality, and 1 yearly visit. We applied a two-sided hypothesis test with a significance set at P < 0.05. All analyses were conducted using SAS Enterprise Guide Version 8.4.

## Results

### Participants

During the years 2015–2023, 1,848,813 individuals in the OCHIN database met our inclusion criteria (Table 1). Overall, the largest proportion of individuals were Hispanic, accounting for 39.0% of the study population, followed by NH White (28.3%), NH Black (16.0%), NH Asian (4.9%), AI/AN (1.1%), NH NHPI/OPI (0.4%), Unknown (8.8%), and NH other/multi-race (1.3%). The total population was slightly more female (56.8%) compared to male (43.1%). The ages included were overall equally represented with ages 9–12 representing 28.2% of the population, 13–18 (32.1%), and 19–26 (39.7%). The majority of the patients were from an urban location (87.4%). The median number of clinical encounters per patient was 3, and most patients (72.7%) lived below 138% of the federal poverty level at some point during the study period. Approximately 65.2% of patients had some form of insurance throughout the duration of the study, followed by uninsured (14.1%) and periods of both insured and uninsured status throughout the duration of the study (20.7%).

**Table 1:** Characteristics of patients eligible for at least one year for HPV vaccination from 2015 to 2023 by race/ethnicity

	Total	AI/AN	Hispanic	NH Asian	NH Black	NH NHPI/OPI	NH Other/Multi-Race	NH White	Unknown
Total (*n*, %)	1,848,813 (100)	20,859 (1.12)	721,829 (39.04)	91,097 (4.92)	296,814 (16.05)	6798 (0.40)	24,196 (1.30)	524,063 (28.34)	163,157 (8.82)
Sex									
Female	1,050,564 (56.82)	12,025 (57.65)	410,254 (56.84)	51,726 (56.78)	166,649 (56.15)	4112 (60.49)	15,020 (62.08)	300,264 (57.3)	90,514 (55.48)
Male	796,390 (43.08)	8803 (42.2)	311,313 (43.13)	39,284 (43.12)	130,045 (43.81)	> 2600 (~ 40)	> 9100 (~ 37)	222,927 (42.54)	72,226 (44.27)
Other/Unknown	1859 (0.1)	31 (0.15)	262 (0.04)	87 (0.1)	120 (0.04)	≤ 10	> 60 (~ 0)	872 (0.17)	417 (0.26)
Age^1^									
9–12	521,869 (28.23)	5501 (26.37)	226,614 (31.39)	25,133 (27.59)	81,789 (27.56)	1812 (26.65)	6687 (27.64)	130,418 (24.89)	43,915 (26.92)
13–18	592,487 (32.05)	7089 (33.99)	238,609 (33.06)	29,615 (32.51)	90,370 (30.45)	2278 (33.51)	8301 (34.31)	163,155 (31.13)	53,070 (32.53)
19–26	734,457 (39.73)	8269 (39.64)	256,606 (35.55)	36,349 (39.9)	124,655 (42)	2708 (39.84)	9208 (38.06)	230,490 (43.98)	66,172 (40.56)
Rurality									
Urban	1,615,900 (87.4)	17,252 (82.71)	669,039 (92.69)	87,696 (96.27)	281,094 (94.7)	6372 (93.73)	20,550 (84.93)	389,309 (74.29)	144,588 (88.62)
Large rural town	150,043 (8.12)	1841 (8.83)	38,491 (5.33)	2175 (2.39)	10,178 (3.43)	254 (3.74)	2481 (10.25)	83,774 (15.99)	10,849 (6.65)
Isolated rural town	80,675 (4.36)	1717 (8.23)	13,987 (1.94)	1136 (1.25)	5361 (1.81)	159 (2.34)	1127 (4.66)	50,288 (9.6)	6900 (4.23)
Missing	2195 (0.12)	49 (0.23)	312 (0.04)	90 (0.1)	181 (0.06)	13 (0.19)	38 (0.16)	692 (0.13)	820 (0.5)
N yearly encounters									
< 1	28,862 (1.56)	267 (1.28)	9824 (1.36)	1269 (1.39)	4901 (1.65)	149 (2.19)	456 (1.88)	9738 (1.86)	2258 (1.38)
1	733,062 (39.65)	7248 (34.75)	263,198 (36.46)	38,752 (42.54)	130,073 (43.82)	3103 (45.65)	8601 (35.55)	204,392 (39)	77,695 (47.62)
2–3	712,270 (38.53)	7858 (37.67)	296,039 (41.01)	37,355 (41.01)	112,624 (37.94)	2547 (37.47)	9000 (37.2)	191,753 (36.59)	55,094 (33.77)
4–7	275,331 (14.89)	3782 (18.13)	115,567 (16.01)	10,883 (11.95)	37,997 (12.8)	792 (11.65)	4240 (17.52)	81,694 (15.59)	20,376 (12.49)
8 +	99,288 (5.37)	1704 (8.17)	37,201 (5.15)	2838 (3.12)	11,219 (3.78)	207 (3.05)	1899 (7.85)	36,486 (6.96)	7734 (4.74)
Percent FPL									
Always < = 138	1,163,906 (62.95)	12,890 (61.8)	483,260 (66.95)	60,939 (66.89)	216,500 (72.94)	4371 (64.3)	14,642 (60.51)	278,935 (53.23)	92,369 (56.61)
Always > 138	188,449 (10.19)	1779 (8.53)	59,980 (8.31)	9766 (10.72)	19,642 (6.62)	652 (9.59)	2970 (12.27)	78,117 (14.91)	15,543 (9.53)
Over and Under	179,807 (9.73)	2118 (10.15)	78,074 (10.82)	9126 (10.02)	20,325 (6.85)	600 (8.83)	2958 (12.23)	57,588 (10.99)	9018 (5.53)
Unknown	316,651 (17.13)	4072 (19.52)	100,515 (13.93)	11,266 (12.37)	40,347 (13.59)	1175 (17.28)	3626 (14.99)	109,423 (20.88)	46,227 (28.33)
Visit coverage									
Always insured	1,205,054 (65.18)	12,315 (59.04)	468,602 (64.92)	64,504 (70.81)	187,272 (63.09)	4421 (65.03)	14,946 (61.77)	347,671 (66.34)	105,323 (64.55)
Always uninsured	260,722 (14.1)	2756 (13.21)	105,007 (14.55)	10,505 (11.53)	44,882 (15.12)	1086 (15.98)	2788 (11.52)	62,307 (11.89)	31,391 (19.24)
Mixed	383,037 (20.72)	5788 (27.75)	148,220 (20.53)	16,088 (17.66)	64,660 (21.78)	1291 (18.99)	6462 (26.71)	114,085 (21.77)	26,443 (16.21)

*AI/AN* American Indian/Alaska Native, *n* number; *NH* Non-Hispanic, *NH NHPI/OPI* Non-Hispanic Native Hawaiian and Other Pacific Islander

Chi-squared analyses showed that all race vs. sociodemographic variable comparisons were significant at *P* < 0.001. Cells with low patient counts are suppressed to protect patient privacy. Additional cells are masked to prevent back-calculation

^1^Age as of first study-eligible encounter

### HPV vaccination initiation and completion rates over time by race/ethnicity

**Fig. 2:**
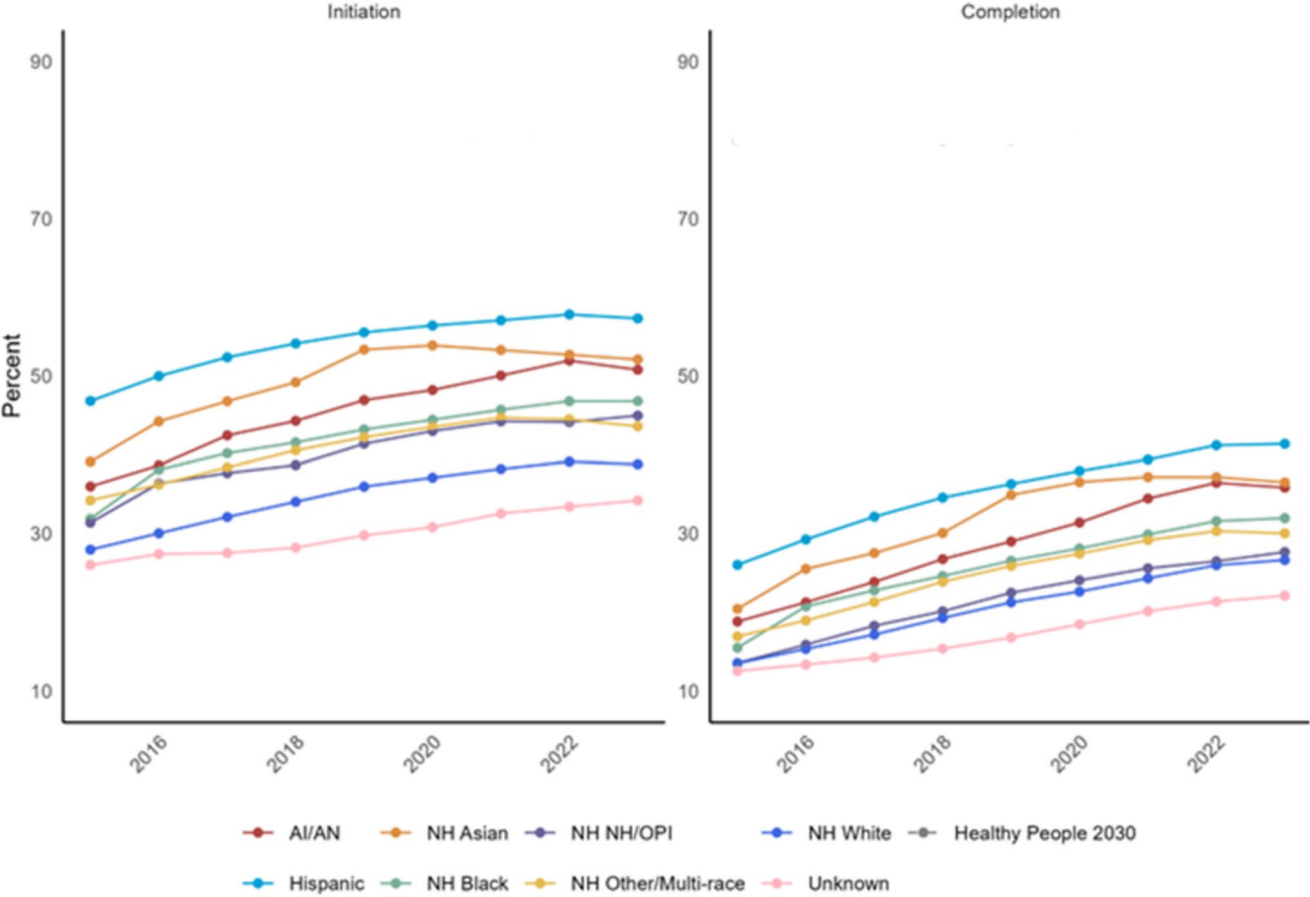
HPV vaccination initiation and completion rates over time for all race/ethnicity groups

Vaccination rates for all racial and ethnic groups remained below the Healthy People 2030 goal of 80% throughout the entire duration of the study period (Fig. 2). The rates of initiation and completion of HPV vaccination between 2015 and 2023 showed notable differences between initiation and completion, with initiation far outpacing completion across all racial categories. All races showed modest but continued uptrends in both initiation and completion during the timeframe of the study. HPV vaccination was the highest among Hispanic individuals and NH White individuals had the lowest rates of vaccination. The NH Asian population showed a peak of both initiation and completion around 2019 and 2020, respectively, with a progressive decline in both initiation and completion from 2019 to 2023. We did not identify a measurable reduction in vaccination rates surrounding the COVID-19 pandemic and peak social distancing orders from 2020 to 2022 in any racial category other than NH Asian individuals.

### HPV vaccination initiation and completion rates over time by race/ethnicity and age group

**Fig. 3:**
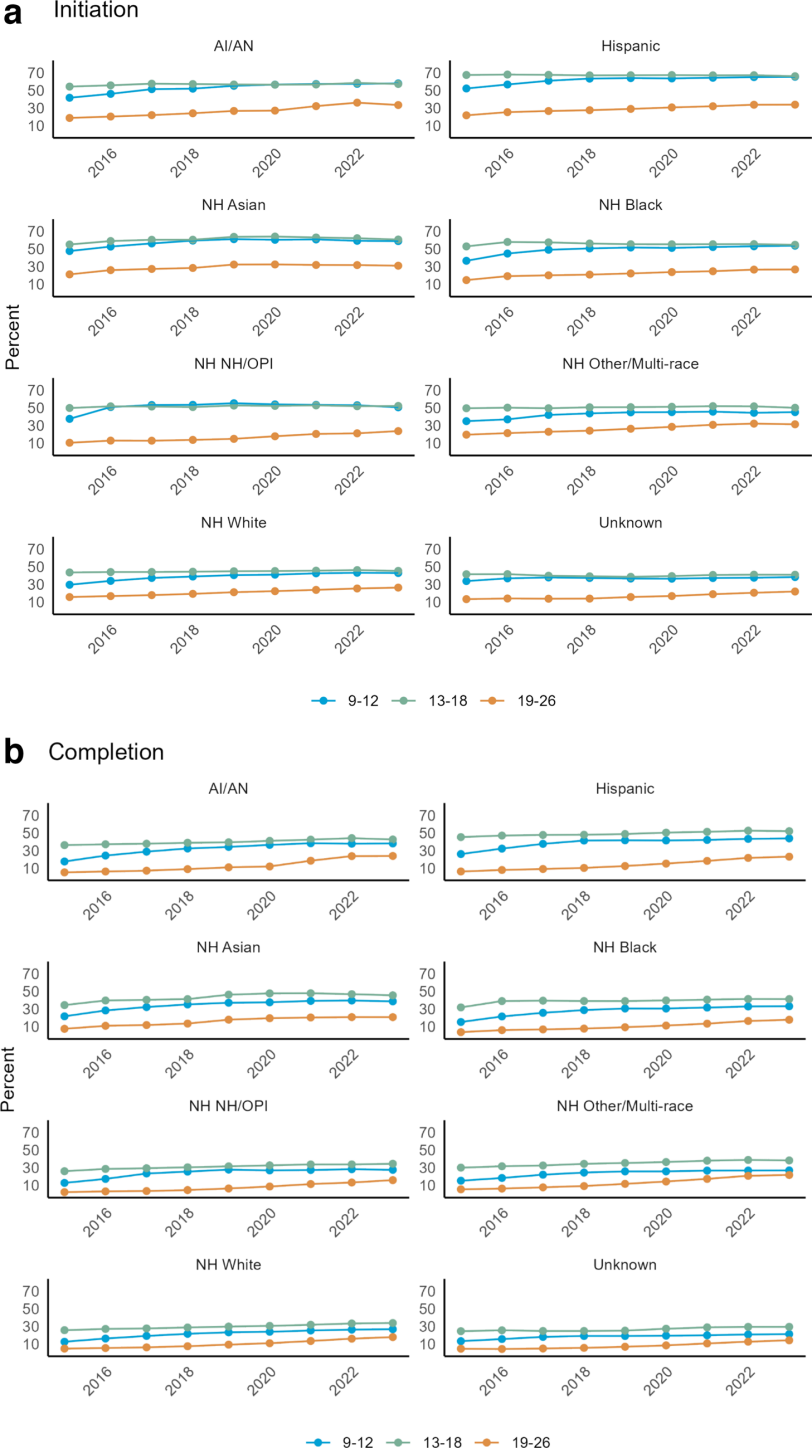
** a** Rates by age group compared across all race/ethnicities for HPV vaccine initiation. ** b** Rates by age group compared across all race/ethnicities for HPV vaccine completion

Initiation and completion rates by age and race demonstrated that all racial groups had increasing rates of HPV vaccine initiation from 2015 to 2023 among the age group of 9–12 in concordance with current vaccination recommendations (Fig. 3a). A similar trend was noted across all races for completion of the vaccine series; however, the 13–18 age group had the highest completion rates across all race/ethnicity groups (Fig. 3b). Patients between the ages of 19 and 26 were the least likely to initiate or complete the HPV vaccination series.

### HPV vaccination initiation and completion rates over time by race/ethnicity and geographic location

**Fig. 4:**
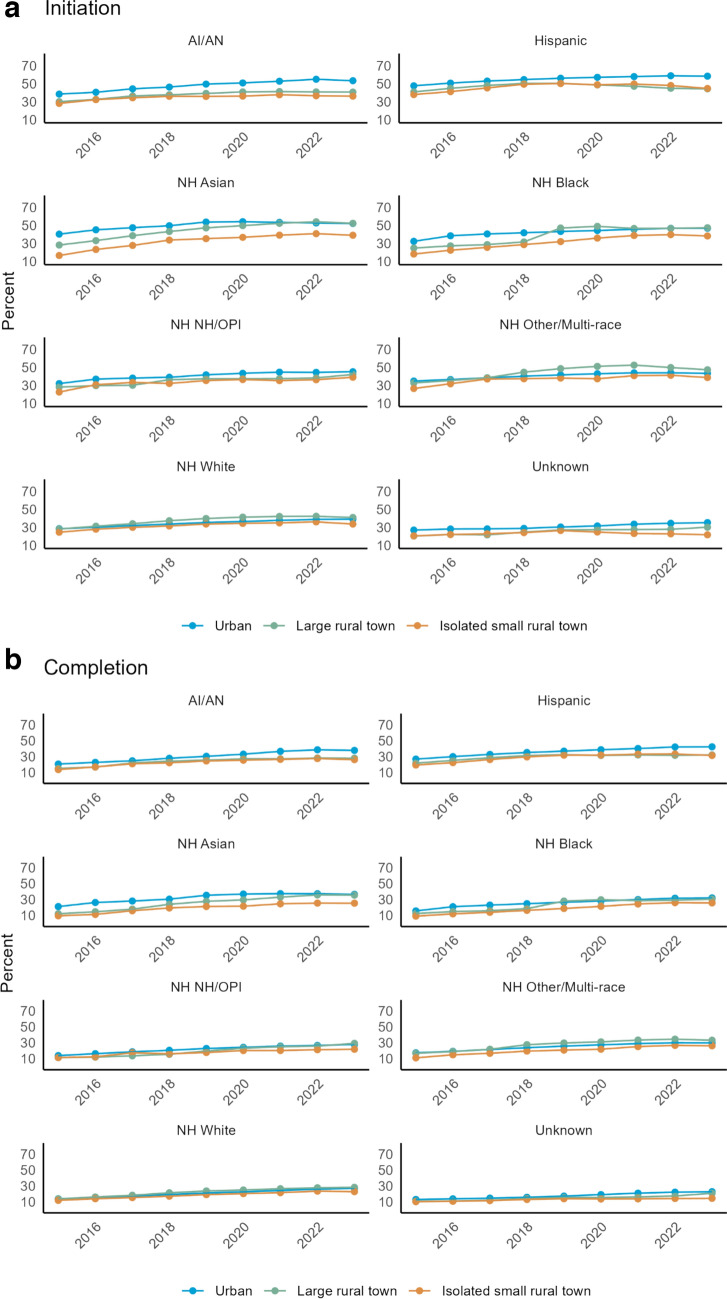
** a** Rates by location compared across all race/ethnicities for HPV vaccine initiation.** b**Rates by location compared across all race/ethnicities for HPV vaccine completion

Initiation and completion by rural–urban commuting area (RUCA) codes and race/ethnicities demonstrated greater percentages in urban settings over large and small isolated rural region (Fig. 4a and b). Individuals living in isolated small rural town RUCA had the lowest rates during this time.

### HPV vaccination rates compared to other recommended childhood vaccines

**Fig. 5:**
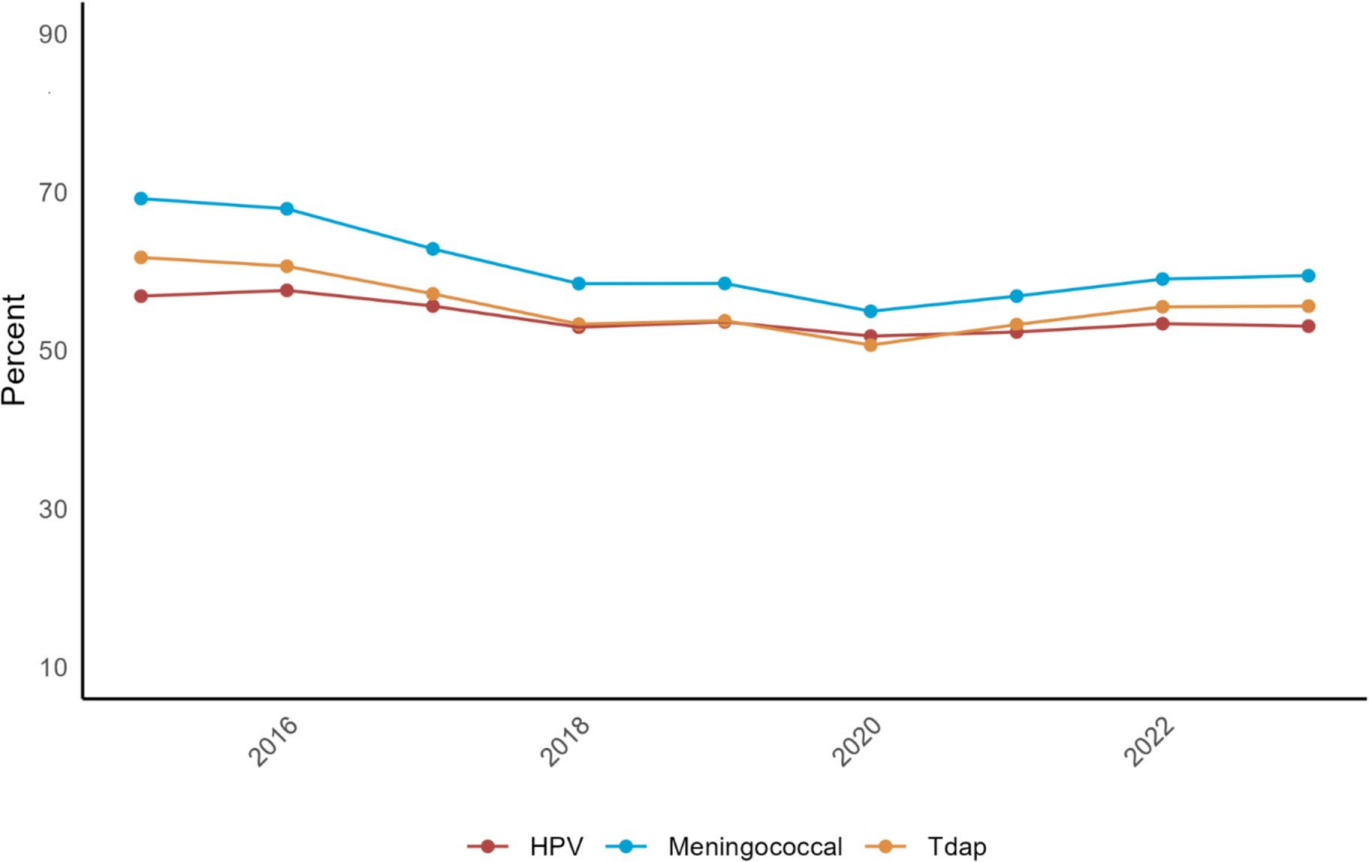
HPV, Meningococcal, and Tdap Vaccine Comparisons for 11-12 year olds only

As the national recommendations for HPV immunization are in conjunction with other routine childhood vaccinations such as Tdap and the Meningococcal vaccine at ages 11–12 years, we compared rates of up-to-date meningococcal and Tdap vaccination and HPV initiation between 2015 and 2023 (Fig. 5). Our results demonstrated that rates of HPV vaccination were lower than those for Tdap (*p* = 0.26) and Meningococcal (*p* = 0.0017) vaccines. We also observed decreasing rates of both Tdap and meningococcal vaccine with a nadir of both in 2020 with gradual recovery through 2023. HPV vaccination, in contrast, did not show a significant nadir coinciding with the COVID-19 pandemic and has remained stagnant at approximately 50%. We also compared rates of these vaccinations by race/ethnicity group with no prominent differences between rates of the individual vaccination uptake noted.

### Odds of HPV vaccine initiation and completion for patients ages 9–26

**Fig. 6:**
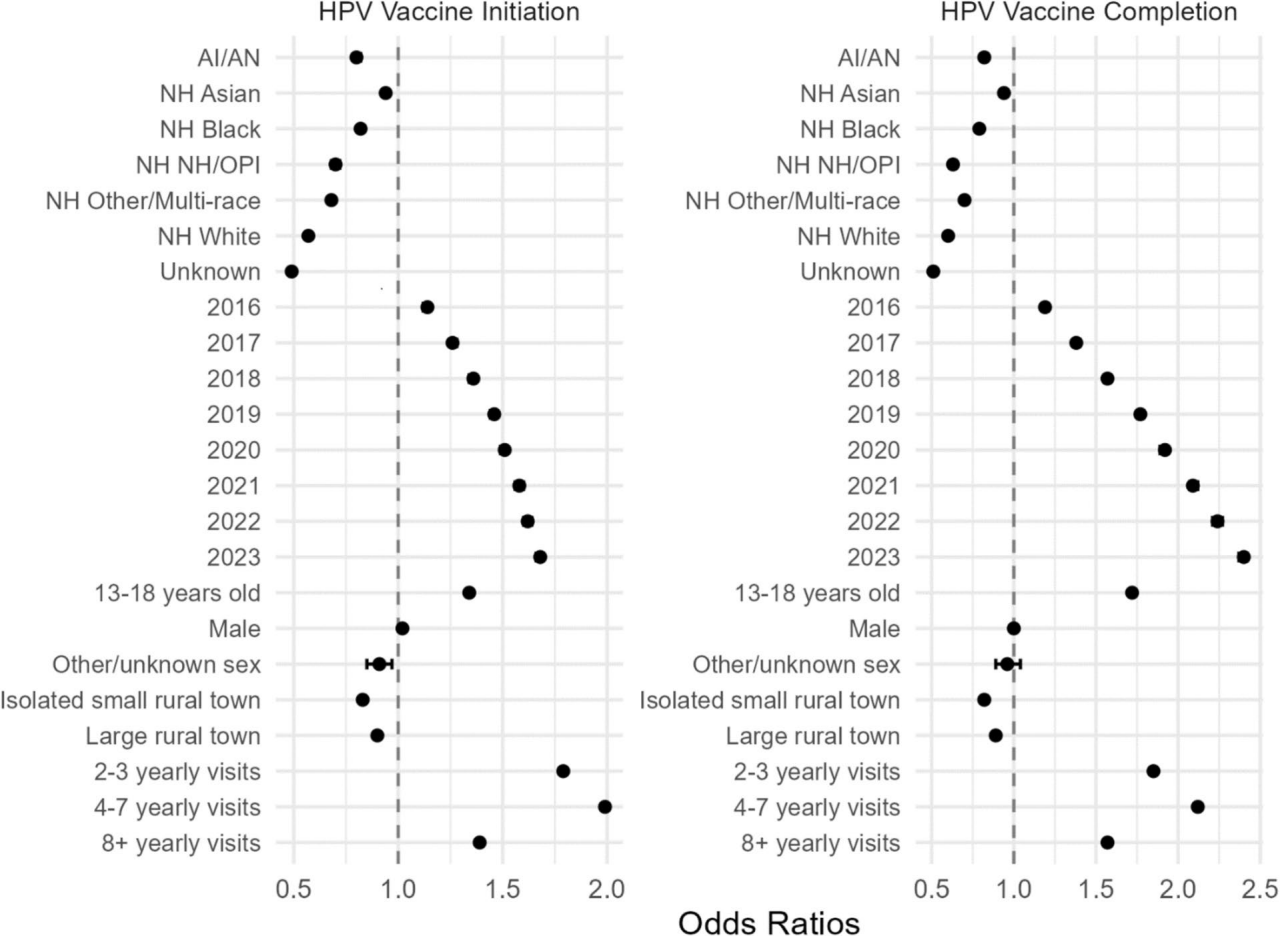
Odds of HPV vaccine initiation and completion for patients eligible for at least 1 year (not including insurance status, FPL, <1 visit)

We investigated the adjusted odds of HPV vaccine initiation and completion among those eligible for at least one year during the study period for several variables (Fig. 6). The most likely group to have HPV vaccine initiation and completion was Hispanic individuals. NHW were least likely to initiate vaccination and complete vaccination OR 0.57 (CI 0.567–0.573) and 0.598 (CI 0.595–0.601) for initiation and completion, respectively. Our analysis showed overall increasing rates of both HPV initiation and completion across the study period; starting with 1.14 (1.12–1.15) and 1.19 (1.18–1.21) odds in 2016; and odds of 1.68 (1.66–1.69) and 2.40 (2.37–2.42) in 2023. The age group of 13–18 years of age were most likely to have initiated (OR 1.34 [1.33–1.35]) and completed the vaccine series (1.72 [1.71–1.73]). Contrary to U.S. trends, we found no statistically significant differences in odds for initiation and completion of the series based on gender. However, the male population did show marginal increased odds of initiation and equal odds of completion compared to females. Individuals with greater than or equal to 2 encounters per year had greater odds of initiating and completing the vaccine, with the greatest odds for individuals having 4–7 yearly encounters. A sub-analysis was performed investigating the odds of vaccine completion among individuals that had received at least one dose of HPV vaccine by age (age 9–10, 11–12, 13–18, 19–26) to examine if earlier age at initiation led to higher odds of completion. This analysis showed that those individuals receiving at least one dose of HPV vaccine between the ages of 9 and 10 were most likely to complete the vaccination series compared to 11–12 (OR 0.71 [0.71–0.72]), 13–18 (OR 0.30 [0.29–0.30]), and 19–26 (OR 0.07 [0.07–0.08]).

## Discussion

This study was performed using a large, diverse, and classically underserved patient cohort from 2015 to 2023 to evaluate rates of HPV vaccination initiation and completion among those eligible for at least one year. Vaccination completion rates for all racial and ethnic groups were well below the Healthy People 2030 goal (80.0%) and 2023 national average (61.4%) ranging from 12.5% to 41.4% throughout the study period [16]. Hispanic individuals had the highest vaccination rates, with median initiation and completion rates of 55.6% and 36.3%, respectively, and non-Hispanic White individuals had the lowest. A recent publication that assessed cervical cancer screening rates in rural and urban populations indicated similar low rates among non-Hispanic White individuals [17]. Individuals ages 13–18 and those with greater than or equal to two clinical visits per year had the greatest odds of completing the HPV vaccination series; however, a sub-analysis of smaller age groupings showed that those who initiated the series between 9 and 10 had the greatest odds of completion relative to the other subgroups.

National trends of initiation and completion of HPV vaccination show increasing rates over time, with no subgroup meeting the national goal of 80%, similar to our data. In 2016, 60.4% (female = 65.1%, male = 37.5%) of adolescents ages 13–17 received at least one dose, and 43.4% (female = 49.5%, male = 37.5%) completed the series [18]. In 2023, 76.8% (female = 78.5%, male = 75.1%) received at least one dose and 61.4% (female = 64.0%, male = 59.0%) completed the series. One notable difference between observed national trends and our findings was the gender difference in HPV vaccination rates. Males lag behind females nationally for both initiation and completion; however, we found that males in the OCHIN population had higher odds of initiation and completion than females, although marginal. The lag between males and females is a likely consequence of the early marketing strategy of the HPV vaccine (FDA-approved for females first and associated with sexual activity), and ongoing strategies have failed to correct this early error. Additionally, HPV vaccination knowledge and awareness efforts have also been female-centered, which has been implicated in the gender disparity [19, 20]. Our data do not show a statistically significant difference between genders which may reflect a more neutral, consistent approach by providers at these clinics. As vaccination of both males and females is crucial, the strategies employed by these community health organizations are deserving of increased attention with the potential to be replicated across the United States.

National guidelines vary in their recommendations for timing of HPV vaccination. ACIP and the USPSTF recommend HPV vaccination at ages 11–12, the American Academy of Pediatrics supports HPV vaccination between ages 9 and 12, and the ACS recommends starting at 9 [19–21]. Our study shows increasing vaccination rates among those ages 9–12 in all racial/ethnic categories over the study period, which aligns with the current national guidelines. Several studies show that earlier initiation of HPV vaccination correlates with higher series of completion rates. Brewer et al. demonstrated that those initiating the HPV vaccine series at 9–10 years compared to those at 11–12 years were significantly more likely to complete the HPV vaccination series for both publicly (70.4% vs. 40.0%) and privately (76.2% vs. 48.1%) insured patients. [6] Results from our study align with these published data and demonstrate that among those that initiate (receive at least one vaccine) between ages 9 and 10 are more likely to have successful rates of completion. We believe that this could be representative of confusion surrounding differing guidelines and possibly hesitancy surrounding HPV vaccination at younger ages. As provider recommendation is a strong correlate to initiation of HPV vaccination, we believe that increased efforts to initiate vaccination between the ages of 9 and 10 will increase the odds of successful completion during the time when the vaccine is known to be most effective. [8, 22, 23] Our data support the ACS recommendation of starting vaccination at age 9 to increase rates of completion. Aligning all national recommendations would decrease confusion regarding recommended timing of vaccination and could lead to higher rates of vaccine completion among all groups.

Reducing vaccine hesitancy and increasing participation in HPV vaccination is critical to the success of achieving the national goal of an 80.0% vaccine completion rate. One strategy to increase HPV vaccination among adolescents has been to bundle vaccinations with other similarly timed recommended childhood vaccines, meningococcal and Tdap [24]. In this study, we examined rates of routine vaccination among OCHIN database for vaccinations received between 11 and 12 years of age and HPV from 9 to 12 years. We found the mean percentages for all vaccinations to be lower than the most recent national data with Meningococcal (60.8%), Tdap (55.7%), and HPV (54.1%). All vaccinations showed a nadir at the time of the COVID pandemic with subsequent uptrend in vaccination after the pandemic with the exception of NH Asian individuals. These data suggest that efforts to reduce vaccine hesitancy for all childhood vaccines are necessary to achieve herd immunity and maximal protection.

Racial and ethnic disparities for HPV-dependent cancers are well described and often associated with the perception of decreased vaccination among minority groups. However, the existing literature examining racial disparities in relation to HPV vaccination is unclear and limited [16–18]. Two systematic reviews showed that vaccine initiation was higher among Black and Hispanic adolescents than non-Hispanic white adolescents; however, vaccine completion was lower [25, 26]. This agrees with several studies that have suggested that differences in HPV vaccination favor minorities [27, 28]. In contrast, other studies have reported that racial minorities were either less likely than NHW to receive HPV vaccination or that there was no difference [29–31]. In this study, we found that NHW was the racial group least likely to initiate or complete HPV vaccination and was comparable only to NH NHPI/OPI, which had the lowest completion rates across all age groups studied. Our study adds to the body of evidence that, among more underserved populations in the OCHIN network, minority populations are more likely than NHW to participate in HPV vaccination. This may reflect increased vaccination efforts in the more traditionally underserved racial minority groups and brings into focus the decreased uptake of vaccination among the NHW population. The NHW population in this high-risk cohort is of particular concern, as they show rates far below all other racial groups both in our study as well as nationally. This highlights the ongoing paradigm shift away from HPV vaccination among the more disadvantaged NHW population, and calls for more dedicated research examining barriers to HPV vaccination at both patient and systems level among this population. [13, 32]

Many communities experienced decreased engagement with preventive healthcare during the COVID-19 pandemic and physical distancing recommendations [33]. Surprisingly, HPV vaccination rates did not decrease overall between 2020 and 2022. In our analysis, NH Asian/Pacific Islander was the only racial group that decreased during this period and did not return to baseline. Asian Americans experienced health challenges during the COVID-19 pandemic that were related to racism and threats of violence, which could have contributed to a lower utilization of services. A 2021 Pew Research Center analysis of the American Community Survey conducted both before and after the pandemic found that English-speaking Asian Americans in 2022 experienced COVID-19 discrimination and “were more likely than other racial or ethnic groups to say they had changed their daily routines due to concerns they might be threatened or attacked” [34]. Researchers of racial and ethnic disparities attribute this to the Anti-Asian racism impacting Asian/PA populations due to COVID-19 indicating a concurrence of health challenges, economic challenges, and increased racism toward Asian communities due to during the COVID-19 pandemic [35].

We also sought to examine differences in HPV vaccination according to geographic location, as this has been found to be a factor in the uptake of HPV vaccination in the existing literature. For example, according to the CDC, individuals living in non-metropolitan areas have lower HPV vaccination rates [15]. However, large meta-analyses have shown mixed effects and large variations between states and regions [36, 37]. This study found that those living in isolated rural towns were less likely to initiate or complete HPV vaccination. However, our population was predominantly urban, which may limit interpretation of our findings. In several studies, examining geographic disparities poverty level was found to be associated with odds of HPV vaccination. For example, in several studies, higher county-level poverty was associated with increased odds of vaccination in contrast to increasing state-level poverty, which was associated with lower odds of vaccination [38–41]. Our study did not show any difference in vaccination by federal poverty level, which may reflect the variations between states and regions, as noted above.

A strength of this study is the large and diverse sample size of underserved, high-risk groups. This allows for greater generalizability to the broader US population beyond the OCHIN network and is reflective of more underserved and socioeconomically disadvantaged patient populations. A limitation of this study is that the population is mainly urban (87.4%). This leads to an underrepresentation of rural communities and individuals residing in those communities. As rural communities are known to have limited access to care, this study may not adequately examine disparities surrounding HPV vaccination in rural areas. Another limitation of this data set includes HPV vaccination rates among the age group of 19–26 as this group is inherently more nomadic and less likely to receive regular healthcare. This makes interpretation of vaccination rates difficult as the data are likely incomplete due to patients either not seeking regular healthcare or utilization of other clinics such as student health.

## Conclusions

HPV vaccination rates in the United States among systemically underserved communities and populations with more significant lifetime risks of HPV-related cancers remain well below the national average and national goals. In this cohort, Hispanic individuals were the most likely to receive HPV vaccination and there was no difference between female and male vaccination rates both of which stand in contrast to national trends. Vaccination initiation between the ages of 9 and 10 demonstrated increased odds of completion which supports the ACS guidelines of initiation at this age range. This study demonstrates that, although disadvantaged groups continue to experience inequity, there are successful trends in increasing HPV vaccination, particularly in community health organizations, that need to be explored.

## Data Availability

For data access: Data can be requested through the OCHIN Data Network formal channels.

## References

[1] Cancers Linked With HPV Each Year. CDC Cancer. httpswwwcdcgov/cancer/hpv/caseshtml?CDC_AAref_Val=httpswwwcdcgov/cancer/hpv/statistics/caseshtm. 2024, Accessed: December 18, 2024.

[2] American Cancer Society I: Steps for Increasing HPV Vaccination in Practice An Action Guide to Implement Evidence-based Strategies for Clinicians. 2024.

[3] Viens LJ, Henley SJ, Watson M et al (2016) Human papillomavirus-associated cancers - United States, 2008–2012. MMWR Morb Mortal Wkly Rep 65:661–666. 10.15585/mmwr.mm6526a1 27387669 10.15585/mmwr.mm6526a1

[4] Markowitz LE, Gee J, Chesson H, Stokley S (2018) Ten years of human papillomavirus vaccination in the United States. Acad Pediatr 18:S3–S10. 10.1016/j.acap.2017.09.014 29502635 10.1016/j.acap.2017.09.014 PMC11331487

[5] Minihan AK, Bandi P, Star J, Fisher-Borne M, Saslow D, Jemal A (2023) The association of initiating HPV vaccination at ages 9–10 years and up-to-date status among adolescents ages 13–17 years, 2016–2020. Hum Vaccin Immunother 19:2175555. 10.1080/21645515.2023.2175555 36748322 10.1080/21645515.2023.2175555 PMC10026883

[6] Saxena K, Kathe N, Sardana P, Yao L, Chen YT, Brewer NT (2023) HPV vaccine initiation at 9 or 10 years of age and better series completion by age 13 among privately and publicly insured children in the US. Hum Vaccin Immunother 19:2161253. 10.1080/21645515.2022.2161253 36631995 10.1080/21645515.2022.2161253 PMC9980633

[7] O’Leary SC, Frost HM (2023) Does HPV vaccination initiation at age 9, improve HPV initiation and vaccine series completion rates by age 13? Hum Vaccin Immunother 19:2180971. 10.1080/21645515.2023.2180971 36892245 10.1080/21645515.2023.2180971 PMC10026893

[8] Ellingson MK, Sheikha H, Nyhan K, Oliveira CR, Niccolai LM (2023) Human papillomavirus vaccine effectiveness by age at vaccination: a systematic review. Hum Vaccin Immunother 19:2239085. 10.1080/21645515.2023.2239085 37529935 10.1080/21645515.2023.2239085 PMC10399474

[9] Meites E, Szilagyi PG, Chesson HW, Unger ER, Romero JR, Markowitz LE (2019) Human papillomavirus vaccination for adults: updated recommendations of the advisory committee on immunization practices. MMWR Morb Mortal Wkly Rep 68:698–702. 10.15585/mmwr.mm6832a3 31415491 10.15585/mmwr.mm6832a3 PMC6818701

[10] Meites E, Kempe A, Markowitz LE (2016) Use of a 2-dose schedule for human papillomavirus vaccination - updated recommendations of the advisory committee on immunization practices. MMWR Morb Mortal Wkly Rep 65:1405–1408. 10.15585/mmwr.mm6549a5 27977643 10.15585/mmwr.mm6549a5

[11] American College of O, Gynecologists' Committee on Adolescent Health Care ACoO, Gynecologists' Immunization ID, Public Health Preparedness Expert Work G: Human Papillomavirus Vaccination: ACOG Committee Opinion, Number 809. Obstet Gynecol. 2020, 136:e15-e21. 10.1097/AOG.0000000000004000 10.1097/AOG.0000000000004000 32732766

[12] Healthy People 2023: Increase the proportion of adolescents who get recommended doses of the HPV vaccine — IID‑08. httpsodphphealthgov/healthypeople/objectives-and-data/browse-objectives/vaccination/increase-proportion-adolescents-who-get-recommended-doses-hpv-vaccine-iid-08/data. 2020, Accessed: December 18, 2024.

[13] Chido-Amajuoyi OG, Talluri R, Wonodi C, Shete S (2021) Trends in HPV vaccination initiation and completion within ages 9–12 years: 2008–2018. Pediatrics. 10.1542/peds.2020-012765 33941585 10.1542/peds.2020-012765 PMC8785751

[14] Kavanagh K, Pollock KG, Cuschieri K et al (2017) Changes in the prevalence of human papillomavirus following a national bivalent human papillomavirus vaccination programme in Scotland: a 7-year cross-sectional study. Lancet Infect Dis 17:1293–1302. 10.1016/S1473-3099(17)30468-1 28965955 10.1016/S1473-3099(17)30468-1

[15] Villarroel MA GA, Lu PJ, Pingali C. (2024) Human Papillomavirus Vaccination Coverage in Children Ages 9–17 Years: United States, 2022. NCHS Data Brief 10.15620/cdc:14559310.15620/cdc:145593 38358336

[16] Pingali C, Yankey D, Chen M et al (2024) National vaccination coverage among adolescents aged 13–17 years - national immunization survey-teen, United States, 2023. MMWR Morb Mortal Wkly Rep 73:708–714. 10.15585/mmwr.mm7333a1 39173168 10.15585/mmwr.mm7333a1 PMC11349384

[17] Borders TF, Thaxton Wiggins A (2024) Cervical cancer screening rates among rural and urban females, from 2019 to 2022. JAMA Netw Open 7:e2417094. 10.1001/jamanetworkopen.2024.17094 38874926 10.1001/jamanetworkopen.2024.17094 PMC11179126

[18] Walker TY, Elam-Evans LD, Singleton JA et al (2017) National, regional, state, and selected local area vaccination coverage among adolescents aged 13–17 years - United States, 2016. MMWR Morb Mortal Wkly Rep 66:874–882. 10.15585/mmwr.mm6633a2 28837546 10.15585/mmwr.mm6633a2 PMC5687818

[19] Patel PR, Berenson AB (2013) Sources of HPV vaccine hesitancy in parents. Hum Vaccin Immunother 9:2649–2653. 10.4161/hv.26224 23982270 10.4161/hv.26224 PMC4162068

[20] Adjei Boakye E, Tobo BB, Rojek RP, Mohammed KA, Geneus CJ, Osazuwa-Peters N (2017) Approaching a decade since HPV vaccine licensure: Racial and gender disparities in knowledge and awareness of HPV and HPV vaccine. Hum Vaccin Immunother 13:2713–2722. 10.1080/21645515.2017.1363133 28853980 10.1080/21645515.2017.1363133 PMC5703403

[21] American Cancer Society I: HPV Vaccines. 2024.

[22] Gilkey MB, Calo WA, Moss JL, Shah PD, Marciniak MW, Brewer NT (2016) Provider communication and HPV vaccination: the impact of recommendation quality. Vaccine 34:1187–1192. 10.1016/j.vaccine.2016.01.023 26812078 10.1016/j.vaccine.2016.01.023 PMC4944755

[23] Oh NL, Biddell CB, Rhodes BE, Brewer NT (2021) Provider communication and HPV vaccine uptake: a meta-analysis and systematic review. Prev Med 148:106554. 10.1016/j.ypmed.2021.106554 33857561 10.1016/j.ypmed.2021.106554

[24] Five Ways to Boost Vaccination Rates> CDC. httpswwwcdcgov/hpv/hcp/vaccination-considerations/boost-rateshtml. 2024, Accessed: December 18, 2024.

[25] Mansfield LN, Vance A, Nikpour JA, Gonzalez-Guarda RM (2021) A systematic review of human papillomavirus vaccination among US adolescents. Res Nurs Health 44:473–489. 10.1002/nur.22135 33860541 10.1002/nur.22135 PMC8248517

[26] Spencer JC, Calo WA, Brewer NT (2019) Disparities and reverse disparities in HPV vaccination: a systematic review and meta-analysis. Prev Med 123:197–203. 10.1016/j.ypmed.2019.03.037 30930259 10.1016/j.ypmed.2019.03.037 PMC6724708

[27] Clarke MA, Coutinho F, Phelan-Emrick DF, Wilbur M, Chou B, Joshu CE (2016) Predictors of human papillomavirus vaccination in a large clinical population of males aged 11 to 26 years in Maryland, 2012–2013. Cancer Epidemiol Biomarkers Prev 25:351–358. 10.1158/1055-9965.EPI-15-0983 26698909 10.1158/1055-9965.EPI-15-0983 PMC4767642

[28] Reiter PL, McRee AL, Pepper JK, Gilkey MB, Galbraith KV, Brewer NT (2013) Longitudinal predictors of human papillomavirus vaccination among a national sample of adolescent males. Am J Public Health 103:1419–1427. 10.2105/AJPH.2012.301189 23763402 10.2105/AJPH.2012.301189 PMC3725571

[29] Chao C, Velicer C, Slezak JM, Jacobsen SJ (2010) Correlates for human papillomavirus vaccination of adolescent girls and young women in a managed care organization. Am J Epidemiol 171:357–367. 10.1093/aje/kwp365 20047978 10.1093/aje/kwp365 PMC3291083

[30] Brewer NT, Gottlieb SL, Reiter PL et al (2011) Longitudinal predictors of human papillomavirus vaccine initiation among adolescent girls in a high-risk geographic area. Sex Transm Dis 38:197–204. 10.1097/OLQ.0b013e3181f12dbf 20838362 10.1097/OLQ.0b013e3181f12dbf PMC3025264

[31] Wei F, Moore PC, Green AL (2013) Geographic variability in human papillomavirus vaccination among U.S. young women. Am J Prev Med 44:154–157. 10.1016/j.amepre.2012.09.061 23332332 10.1016/j.amepre.2012.09.061 PMC3552249

[32] Boersma P, Black LI: Human Papillomavirus Vaccination Among Adults Aged 18–26, 2013–2018. NCHS Data Brief. 2020:1–8. 32487295

[33] Sonawane K, Garg A, Meissner EG et al (2024) Human papillomavirus vaccination among young adults before and during the COVID-19 pandemic. JAMA Netw Open 7:e2356875. 10.1001/jamanetworkopen.2023.56875 38376844 10.1001/jamanetworkopen.2023.56875 PMC10879942

[34] Ruiz N, Im, C., Tian, Z.: Discrimination Experiences Shape Most Asian Americans’ Lives, Stereotypes of Asians in the U.S. as foreigners and a model minority drive discrimination. November 30, 2023

[35] McGarity-Palmer R, Saw A, Horse AJY, Yi SS, Tsoh J, Takeuchi D (2024) Profiles of a COVID-19 syndemic: anti-Asian racism, economic challenges, and mental and physical health. J Racial Ethn Health Disparities 11:300–312. 10.1007/s40615-023-01519-3 36692660 10.1007/s40615-023-01519-3 PMC9872729

[36] Promotion OoDPaH: Increase the proportion of adolescents who get recommended doses of the HPV vaccine — Data - Healthy People 2030. 2020.

[37] Do EK, Rossi B, Miller CA et al (2021) Area-level variation and human papillomavirus vaccination among adolescents and young adults in the United States: a systematic review. Cancer Epidemiol Biomarkers Prev 30:13–21. 10.1158/1055-9965.EPI-20-0617 33008874 10.1158/1055-9965.EPI-20-0617 PMC8108385

[38] Henry KA, Swiecki-Sikora AL, Stroup AM, Warner EL, Kepka D (2017) Area-based socioeconomic factors and human papillomavirus (HPV) vaccination among teen boys in the United States. BMC Public Health 18:19. 10.1186/s12889-017-4567-2 28709420 10.1186/s12889-017-4567-2 PMC5513319

[39] Lu PJ, Yankey D, Fredua B et al (2019) Association of Provider Recommendation and Human Papillomavirus Vaccination Initiation among Male Adolescents Aged 13–17 Years-United States. J Pediatr 206(33–41):e31. 10.1016/j.jpeds.2018.10.034 10.1016/j.jpeds.2018.10.034 PMC6487492 30448270

[40] Nanagas VC, Stolfi A, Nanagas MT, Eberhart GM, Alter SJ (2016) Adolescent male human papillomavirus vaccination. Glob Pediatr Health. 10.1177/2333794X16642373 27336012 10.1177/2333794X16642373 PMC4905155

[41] Pruitt SL, Schootman M (2010) Geographic disparity, area poverty, and human papillomavirus vaccination. Am J Prev Med 38:525–533. 10.1016/j.amepre.2010.01.018 20409501 10.1016/j.amepre.2010.01.018 PMC3259737

